# Preclinical imaging methods for assessing the safety and efficacy of regenerative medicine therapies

**DOI:** 10.1038/s41536-017-0029-9

**Published:** 2017-10-19

**Authors:** Lauren Scarfe, Nathalie Brillant, J. Dinesh Kumar, Noura Ali, Ahmed Alrumayh, Mohammed Amali, Stephane Barbellion, Vendula Jones, Marije Niemeijer, Sophie Potdevin, Gautier Roussignol, Anatoly Vaganov, Ivana Barbaric, Michael Barrow, Neal C. Burton, John Connell, Francesco Dazzi, Josefina Edsbagge, Neil S. French, Julie Holder, Claire Hutchinson, David R. Jones, Tammy Kalber, Cerys Lovatt, Mark F. Lythgoe, Sara Patel, P. Stephen Patrick, Jacqueline Piner, Jens Reinhardt, Emanuelle Ricci, James Sidaway, Glyn N. Stacey, Philip J. Starkey Lewis, Gareth Sullivan, Arthur Taylor, Bettina Wilm, Harish Poptani, Patricia Murray, Chris E. P. Goldring, B. Kevin Park

**Affiliations:** 1 Department of Cellular and Molecular Physiology, University of Liverpool, Liverpool, UK; 2 Centre for Preclinical Imaging, University of Liverpool, Liverpool, UK; 3 Department of Molecular and Clinical Pharmacology, University of Liverpool, Liverpool, UK; 4 Medical Research Council Centre for Drug Safety Science, University of Liverpool, Liverpool, UK; 5 College of Health Science, University of Duhok, Duhok, Iraq; 6 GlaxoSmithKline, David Jack Centre for Research and Development, Ware, UK; 7 Leiden Academic Centre for Drug Research, Leiden University, Leiden, Netherlands; 8 SANOFI Research and Development, Disposition, Safety and Animal Research, Alfortville, France; 9 Department of Biology, University of Konstanz, Konstanz, Germany; 10 Department of Biomedical Science, University of Sheffield, Sheffield, UK; 11 Department of Chemistry, University of Liverpool, Liverpool, UK; 12 iThera Medical, Munich, Germany; 13 Centre for Advanced Biomedical Imaging, University College London, London, UK; 14 Department of Haemato-Oncology, King’s College London, London, UK; 15 Takara Bio Europe AB, Gothenburg, Sweden; 16 Roslin Cells, University of Cambridge, Cambridge, UK; 17 Medicines and Healthcare Products Regulatory Agency, London, UK; 18 ReNeuron Ltd, Pencoed Business Park, Pencoed, Bridgend, UK; 19 GlaxoSmithKline, Medicines Research Centre, Gunnels Wood Road, Stevenage, UK; 20 Paul Ehrlich Institut, Langen, Germany; 21 Institute of Veterinary Science, University of Liverpool, Liverpool, UK; 22 Phenotox, Macclesfield, UK; 23 UK Stem Cell Bank, Division of Advanced Therapies, National Institute for Biological Standards Control, Medicines and Healthcare Products Regulatory Agency, London, UK; 24 Medical Research Council Centre for Regenerative Medicine, University of Edinburgh, Edinburgh, UK; 25 Department of Biochemistry, Institute of Basic Medical Sciences, University of Oslo, Oslo, Norway; 26 Norwegian Center for Stem Cell Research, Blindern, Oslo, Norway; 27 Institute of Immunology, Oslo University Hospital-Rikshospitalet, Nydalen, Oslo, Norway; 28 Hybrid Technology Hub—Centre of Excellence, Institute of Basic Medical Sciences, University of Oslo, Blindern, Oslo, Norway

## Abstract

Regenerative medicine therapies hold enormous potential for a variety of currently incurable conditions with high unmet clinical need. Most progress in this field to date has been achieved with cell-based regenerative medicine therapies, with over a thousand clinical trials performed up to 2015. However, lack of adequate safety and efficacy data is currently limiting wider uptake of these therapies. To facilitate clinical translation, non-invasive in vivo imaging technologies that enable careful evaluation and characterisation of the administered cells and their effects on host tissues are critically required to evaluate their safety and efficacy in relevant preclinical models. This article reviews the most common imaging technologies available and how they can be applied to regenerative medicine research. We cover details of how each technology works, which cell labels are most appropriate for different applications, and the value of multi-modal imaging approaches to gain a comprehensive understanding of the responses to cell therapy in vivo.

## Introduction

Cell-based regenerative medicine therapies (RMTs) and their translation to clinical application are now a major focus of research and are likely to play a key role in future clinical practice. Broadly, cell-based RMTs encompass various cell types, including stem cells, stromal cells, and macrophages and have the potential to treat many diseases, including neurodegenerative and musculoskeletal disorders.^1^ Many RMTs have shown great promise in preclinical studies for various diseases, including kidney^2^ and liver diseases,^3^ type I diabetes and myocardial infarction^4^; however, success in the clinical setting is limited, with only a small panel of fully approved RMTs available to patients, such as dermal reconstruction, or repair of orthopaedic defects.^5^ The slow translation of RMTs from bench to bedside is primarily due to the lack of convincing data on the safety of RMTs, in addition to uncertainties on the true efficacy and mode of action of the cell therapy.^6^ The importance of acquiring convincing safety and efficacy data in preclinical models before applying such therapies in man is underscored by the disastrous outcomes of bioengineered tracheal transplantation, a procedure that was applied in man before being shown to be safe or effective in animals.^7^ Commercial stem-cell clinics around the world can now use autologous cellular therapies outside the experimental clinical trial settings endangering patient’s health.^8^ A clear example happened in three patients in the US whom clinically received intravitreal injections of autologous adipose tissue-derived “stem cells” and developed severe bilateral visual loss.^9^

The main concerns regarding translation of cell-based RMTs to the clinic are:

1. Tumourigenicity—Pluripotent stem cell-based RMTs are a particular concern due to the propensity of these cells to from teratomas and/or teratocarcinomas; it is important for the tumourigenicity of these cell-based RMTs to be assessed in animal models before being used in the clinic.
2. Immunogenicity—RMTs consisting of allogeneic cells have the potential for evoking an immune reaction in the host; this needs to be managed with respect to the function of the therapy before the RMT is translated to the clinic.
3. Efficacy—The RMT must be proven to have greater efficacy compared to standard therapies for treating a particular disease.
4. Mechanisms of action—It is important to fully understand why the RMT is having a beneficial effect in order to understand whether the cells themselves are therapeutic, or their derived factors.
5. Risk:Benefit ratio—All of the above points need to be considered with the risk:benefit ratio in mind. For example, a small risk of tumourigenicity is likely to be more acceptable if it is being used to treat a life-threatening disease with no alternative treatment (high benefit), than if the RMT is being used to treat a condition that is not life-limiting and/or only provides a modest advantage over current treatments (low benefit).

Relevant animal models, where available, are essential to gain a better understanding of both the efficacy and the safety of cell-based RMTs. Current methods generally rely on histological analysis of tissues post-mortem.^10^ This approach requires many experimental animals to be sacrificed at multiple time points in order to gain a comprehensive insight into in vivo processes following administration of the RMT. Importantly, it does not allow researchers to monitor individual animals over the course of their treatment. This need can be addressed by developing non-invasive imaging methods that can monitor the response of each animal longitudinally.^11^

Preclinical imaging encompasses several different imaging modalities, some of which are only suitable for imaging small animals, and others that can be used in large animals and in the clinic.^12^ Modalities which can be universally applied include magnetic resonance imaging (MRI) and nuclear imaging. Other modalities, such as optical and whole-body optoacoustic imaging, can only be used in small animals, but are nevertheless invaluable because they allow the whole-body biodistribution of the cells to be monitored over the long-term using genetic reporters; this is not currently possible in the clinical setting.

We aim to provide a review of preclinical imaging with a particular focus on assessing the safety, efficacy, and mechanisms of action of RMTs. There are several different imaging modalities available in preclinical research, but this review will focus on the four main modalities, which are: optical (fluorescence and bioluminescence imaging (FLI; BLI)), MRI, nuclear imaging, and optoacoustic imaging.

## Preclinical imaging and cell labelling

### Imaging modalities

Optical imaging is a commonly used modality, as it can provide fast, high-throughput, whole-body imaging^13^ (see Box 1, Table 1). Transplanted cells containing fluorescence or (bio)luminescence, either as a result of directly labelling the cells with probes (see Box 2) or by introducing reporter genes (see Box 3), can be tracked using optical imaging, thus allowing the monitoring of cell biodistribution and tumour formation. Disadvantages of optical imaging include low penetration depth, poor spatial resolution, and poor quantification capabilities.^13^

**Table 1:** Summary of the features of the four most commonly used imaging modalities in preclinical research

Imaging modality	Features	Cell tracking	Other applications for regenerative medicine
Optical Imaging: (Bio)luminescence and fluorescence Imaging (BLI; FLI)	Spatial resolution: 2–5 mmTemporal resolution**:** seconds to minutesPenetration depth**:** < 1 cm for fluorescence, 1–2 cm for bioluminescenceSafety**:** completely safe	Cells transduced with reporter gene can be tracked; the signal disappears with cell death (no false-positives). Good for tracking cell fate.	Tracking of biological processes and molecular pathways such as cell signalling.Gene transfer efficiency in gene therapy preclinical research.^128^Tumour imaging.^92,129^Cell differentiation.^74^
		Semi-quantitative method. Output measured in relative light units (RLUs), which vary between different luminometers.	
		Good cell tracking with fluorescent quantum dots, however signal weakens with cell division and quantum dots from dead cells can be phagocytosed by macrophages and yield false positives—not suitable for tracking cell fate.^127^	
		Alternatively, persistent luminescent particles have excellent signal to noise ratio, and reduced tendency to be released from cells and so can be used to track cells for longer periods of time than most other optical probes.^108^	
Magnetic resonance imaging (MRI)	Spatial resolution: 40–100 umTemporal resolution**:** minutes to hoursPenetration depth**:** no limitSafety: completely safe	Cells can be labelled with superparamagnetic iron oxide nanoparticles (SPIONs) or paramagnetic metal chelates.	Oncology (tumour growth, perfusion, ablation and oxygenation).^131^Cardiology (heart perfusion).^132^Musculoskeletal tissue structures.^133^
		Magnetic reporters can also be used to track cells, but lack sensitivity.^55,130^	
Nuclear Imaging: PET and SPECT	Spatial resolution: 1–2 mmTemporal resolution**:** seconds to minutesPenetration depth**:** unlimitedSafety**:** there are some safety concerns over the use of radioactive tracers, however doses are very low and the risks are carefully monitored	Cells can be labelled with tracers for short-term tracking, for example ^111^In (SPECT) or ^18^F-Fluoro-Deoxyglucose (PET).	PET or SPECT provide high sensitivity, which is an advantage for tracking anatomical localization of stem cells and nuclear imaging using reporter genes permits long-term engraftment studies.
		SPECT and PET reporter gene imaging use the principle of interactions between an exogenous probe and the protein produced by the reporter gene. There are predominantly three genes: herpes simplex virus type 1 thymidine kinase (HSV1-tk), dopamine type 2 receptor (D2R), and, sodium/iodide symporter (NIS).^97^	
Photoacoustic Imaging	Spatial resolution: 20–300 μmTemporal resolution**:** seconds to minutesPenetration depth**:** 4–5 cmSafety**:** completely safe	Can image cells labelled with gold nanorods^30^ or carbon nanotubes^134^, or cells expressing NIR reporter genes.^117^ Can image down to 10,000 cells^30^ and can quantify cell numbers.^135^	Excellent tumour imaging.^22^Functional imaging of some organs/tissues (using either endogenous or exogenous contrast).^136^Imaging of 3D scaffolds.^81^

MRI provides excellent anatomical information with unlimited tissue penetration depth^14^ (see Box 1, Table 1), allowing detailed structural examination of organs before and after RMT administration. Additionally, cells labelled with paramagnetic or superparamagnetic agents (see Box 2) or over-expressing magnetic resonance (MR) reporter genes such as ferritin, tyrosinase, or β-galactosidase (see Box 3) can be tracked using MRI, although most MR reporters have been shown to have limited efficacy.^15^ The detailed structural information obtained from MR images allows the biodistribution of labelled cells to be attributed to specific organs with far greater accuracy and spatial resolution than with optical imaging. However, it is difficult to track cells in regions with inherently variable MRI contrast, such as the lungs, bone, gut, and spleen.^16^ Because cell tracking via MR is dependent on the effect the labelling agent has on water proton signal (T_1_/T_2_ relaxation), the observed contrast is not always easily discriminated from other pathological processes. For example, labelling with iron oxides reduces T_2_/T_2_^*^ signal, an effect that is also seen in areas of haemorrhage or in iron overload diseases such as hemosiderosis. Unlike iron oxides, gadolinium chelates give a positive T_1_ signal but at a lower sensitivity and greater likelihood of cell toxicity, making it less suitable for biodistribution studies. None of these MR methods can be directly correlated with cell number and thus provide no quantifiable metrics of cell distribution. Perfluorocarbons yield signal that originates unequivocally from the labels and that can be quantitated and directly correlated to the number of cells in the tissue, but requires specialised coils and still suffers from a relatively low sensitivity, particularly at clinical field strengths.^17^

Nuclear imaging makes use of radioactive probes to produce images of physiological or functional significance from within the body.^18, 19^ There are two major nuclear imaging modalities used in both preclinical and clinical practice: PET and SPECT. Cells directly labelled with radionuclides such as Indium-111 (^111^In), Zirconium-89 (^89^Zr), and Technetium-99m (^99^mTc) (see Box 2) or transduced with nuclear reporter genes (see Box 3) can be tracked with very high sensitivity; however the application of nuclear imaging is limited due to the use of short-lived radioisotopes, and the potential negative effects on the health of the therapeutic cells.^20^

Optoacoustic imaging is a relatively new imaging modality which has become more commonly used in recent years. Optoacoustic imaging relies on strong light absorbers, which can be endogenous or exogenous molecules or probes (see Box 1, Table 1). Endogenous biological contrasts include oxy-haemoglobin and deoxy-haemoglobin, melanin, water, and lipids,^21^ and are valuable for imaging vasculature, oxygenation status, and tumours.^22^ However, it is the use of exogenous contrast that is of particular interest to RMT applications. A commercially available optoacoustic imager called ‘multispectral optoacoustic tomography’ (MSOT) is capable of imaging at multiple wavelengths.^23^ By detecting acoustic waves, MSOT is able to overcome the scattering of emitted light which usually limits the detection depth of many optical imaging methods, thus permitting imaging depths of several centimetres, and allowing the whole-body imaging of a mouse.^24^ Importantly, by illuminating tissues at multiple wavelengths, the signals from different absorbers can be spectrally unmixed, allowing the differential identification of multiple absorbers at once. For cell tracking purposes, cells can be labelled with probes that absorb within the near-infrared (NIR), such as gold nanorods^25^ or carbon nanotubes (see Box 2), or can be labelled with reporter genes encoding NIR fluorescent proteins (see Box 3). Moreover, optoacoustic imaging can be performed in real time, allowing quantitative assessment of organ function,^26^ thus enabling efficacy studies in addition to cell tracking. Even though this imaging technology is relatively new it has recently been clinically used in oncology,^27^ human vasculature^28^ and inflammatory response.^29^

### Imaging: how does it work?

**Optical Imaging: Luminescence and Fluorescence** Optical imaging (i.e. bioluminescence or fluorescence imaging) of cell-based RMTs involves the detection of emitted light from cells expressing an appropriate reporter gene or labelled with a molecular probe.^92^ The reporter gene required for bioluminescence imaging encodes a luciferase enzyme which catalyses the oxidation of an exogenously administered substrate, and results in the release of a photon.^13^ For fluorescence imaging, light of a particular wavelength is emitted by fluorescent molecular probes or by fluorescent proteins encoded by reporter genes following their excitation with a particular wavelength of light.^92^ The excitation and emission light can occur in the visible part of the spectrum (400–700 nm) or in the near infrared (800–1900 nm),^93^ although the longer wavelengths are better suited for in vivo imaging due to the optical window (see box 4). The detection of the light signal is performed by a cooled charge-coupled device (CCD) camera.^93^ The CCD is a photon detector that operates under a very low temperature in order to eliminate any background noise, allowing a small number of photons to be detected,^92^ thus increasing sensitivity of detection.

**Magnetic Resonance Imaging (MRI)** MRI uses a strong static magnetic field to force the alignment of the spin moments of water protons in the subjects’ body relative to the magnetic field. When a radiofrequency (RF) pulse is applied, the spins are forced to a higher but unstable energy level. As soon as the radiofrequency pulse is turned off, these spins realign with the main magnetic field, releasing energy which is then detected by radiofrequency coils. The time taken to realign with the magnetic field is called the spin-lattice, or T_1_ relaxation. At the same time, some energy is dissipated where the spins are dephased due to local magnetic field inhomogeneities. The time taken for water protons to dephase is called the spin–spin relaxation, or T_2_ relaxation. Different tissues exhibit specific T_1_/T_2_ relaxation times, which results in the characteristic contrast on greyscale images after image reconstruction. This contrast in signal intensity allows differentiation between various normal and pathological tissues, and those containing contrast agents, based on their spin relaxation properties^94^ Iron-based agents and reporters (SPIONs and reporters based on ferritin, transferrin or other genes related to iron metabolism) reduce transverse (T_2_/T_2_^*^) relaxation, leading to hypointense contrast (darkening) in the areas where the labelled cells are present. Paramagnetic-based agents such as those based on gadolinium chelates shorten T_1_ relaxation, leading to positive (bright) signal in T_1_-weighted images. In perfluorocarbon-based imaging, the spin alignment and relaxation processes described above apply to fluorine atoms instead of water atoms. Because soft tissue is essentially absent from fluorine, there is negligible background and all signal is generated by the fluorine-containing agent. A fluorine coil is required, and images are usually overlaid with that of water proton imaging to obtain an anatomical reference.

**Positron emission tomography and single photon emission computed tomography** Positron emission tomography (PET) or single photon emission computed tomography (SPECT) are nuclear imaging modalities and utilize molecules containing radioactive atoms, which are structurally unstable and strive to achieve greater stability by releasing energy/particles through radioactive decay. Different types of radiation with different frequencies and energy may be released from radionuclides, penetrating short or long distances in the tissue.^95^ This ionizing energy is then detected by PET or SPECT detectors and the original location of the signal can be back-projected to generate an image. PET tracers, such as fluorine-18 or zirconium-89, emit small particles called positrons, which begin to lose kinetic energy after they have been produced, until they eventually undergo a process called annihilation. This process involves the collision of positrons with nearby electrons within the body, and typically occurs within 1–2 mm of the original site of decay, resulting in the release of two high energy photons, moving in opposite directions.^96^ These coincident photons are then measured by collinearly aligned detectors, which provides the much higher sensitivity in PET imaging.^97^ In contrast, SPECT imaging uses one or more rotating gamma cameras in order to detect gamma rays (photons), which are directly emitted by SPECT tracers such as indium-111 and technetium-99.^98^

**Optoacoustic Imaging** A short pulse of non-ionising laser energy is applied to the sample, which can potentially absorb the energy and convert some of it into heat. The rise in temperature causes thermoelastic expansion and subsequent relaxation, resulting in a spherical acoustic pressure wave which can be detected as an ultrasound wave. Different tissues of the body will have different absorption, thermal, and elastic properties, and so the resulting ultrasound waves will reach the detector at different times and with different amplitudes depending on the depth and characteristics of their tissue of origin. Contrast is driven by strong light absorbers—such as haemoglobin, melanin, and lipids—that are naturally present in the body, and exogenous absorbers—such as gold nanorods, carbon nanotubes, some fluorescent proteins, and some organic dyes—can potentially be introduced to increase contrast. A optoacoustic image can then be generated by reconstructing the detected acoustic signals, using their magnitude and arrival times at the detector to determine their location of origin.^99^ Optoacoustic detectors arrays come in two varieties: linear and tomographic. Linear arrays have the advantage of comparative ease in adaptation of standard ultrasound imaging systems to include optoacoustic imaging, as ‘off-the-shelf’ linear array ultrasound transducers can be used in combination with suitable lasers to generate optoacoustic images.^100^ Alternatively, tomographic arrays have also been developed for optoacoustic imaging.^101^ As compared to linear array reconstruction, tomographic reconstructions produce more faithful images of anatomy since signals are detected from multiple angles. Scanning occurs by translating the animal through the imaging plane ^102^ similar to MRI, PET and CT—which eliminates user variability induced by using a handheld device or approaches in which a detector comes into direct contact with the skin. The disadvantage is the need to use custom-made transducers, which come at greater cost and are more complex. As with traditional ultrasound imaging, optoacoustic imaging is performed with detectors that vary in centre frequency. Low frequency transducers (e.g., 1–10 MHz) allow deeper tissue penetration (e.g., on the order of several centimetres) ^103^ but have characteristic lower spatial resolution (e.g., down to 80 um),^104^ while high frequency transducers (e.g., 15–40 MHz) have limited tissue penetration (e.g., less than 1.0 cm) but greater spatial resolution.^105^

### Cell labelling

**Fig. 1:**
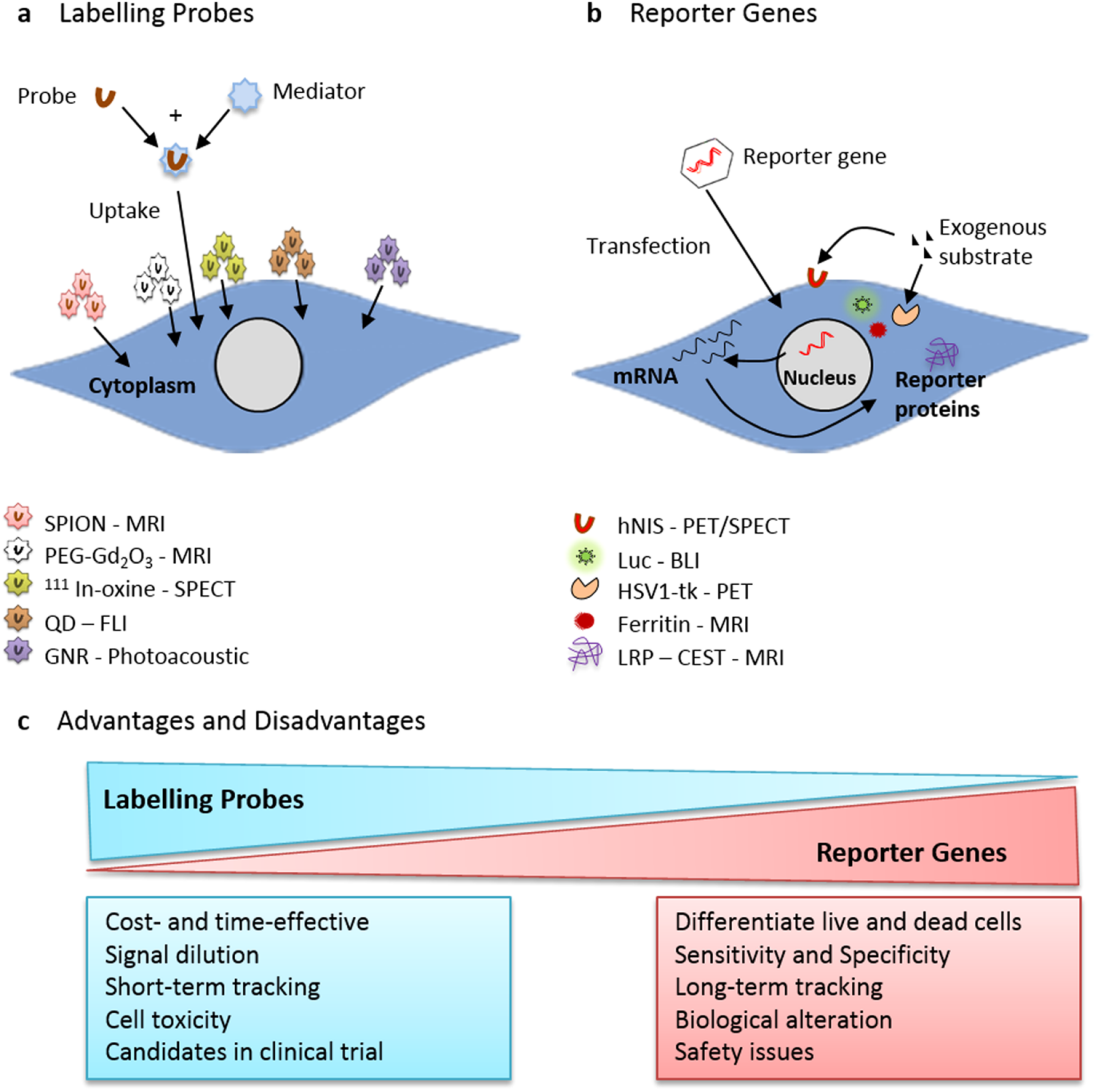
Diagram of the two classes of cell labelling methods, labelling probes and reporter genes, and examples of the labels used for each. **a** Labelling cells with probes involves the uptake of exogenous probes e.g. SPION, ^111^In-oxine, QD, GNR, directly in to the cytoplasm of cells. **b** Reporter gene labelling requires the introduction of foreign DNA into the cell’s DNA, to express a reporter protein. The reporter protein either generates signal using endogenous substrates (e.g., ferritin), or it interacts with an exogenous substrate/detectable probe (e.g., luciferase, HSV1-tk, human sodium iodide symporter (hNIS))

As indicated above, there are two broad categories of cell labels: labelling probes and reporter genes. Labelling probes, also known as direct labels, are required to be taken up by the cells (see Box 2, Fig. 1), whereas the use of reporter genes requires genetic modification of the cells (see Box 3, Fig. 1).

Probes used for cell labelling can produce a very strong signal due to high cellular uptake^30^; however, they suffer from the disadvantage that, when imaging, it is the probe, and not the cell itself, which is being imaged.^31^ This presents a problem for false positive results in cell tracking if the probe is released from the cell of interest and taken up by host cells.^32^ Moreover, when cells labelled with probes divide, the probes within the cell are distributed between the daughter cells, resulting in signal dilution.^31^ Therefore, when monitoring tumour growth, the probe can no longer be detected following several cell divisions.

With the exception of luciferase enzymes, reporter genes (e.g., MR and optoacoustic reporters) tend to produce weaker signals than imaging probes,^33^ with the limiting factors including substrate biodistribution, background uptake and clearance, and/or the expression levels of the reporter gene that can be achieved in a given cell type.^34^ Moreover, some reporter genes, such as the nuclear reporter gene HSV1-tk can generate an immune response in the host, thus limiting the potential for long-term imaging.^35^ The advantage of reporter genes, however, is that the genetic modification required to label the cells is passed onto daughter cells during cell division, so that the signal intensity increases as the cells proliferate, and signals are only lost when the cells die.^36^

The ideal cell tracking agent should:

(I) be non-toxic to the cell and should not change the cell’s phenotype, function, or differentiation potential;
(II) be easily taken up by the cell and should remain in the desired location, either intracellular or membrane-bound;
(III) emit a strong signal for easy detection following administration;
(IV) allow for quantification of cell number;
(V) enable live and dead cells to be distinguished;
(VI) permit the identification of the cell’s metabolic and differentiation status. However, this is challenging and so far has only been achieved using reporter genes under the control of cell-specific promoters.^37^

If using fluorescence or optoacoustic imaging, the label should absorb light maximally within the NIR wavelength range (see Box 4), as it is within this range that the absorption of endogenous pigments, such as haemoglobin, melanin and fat are at a minimum. This allows light to penetrate deeper into the tissue, and signals from cell labels can be detected from deeper within the animal’s body.

**Fig. 2:**
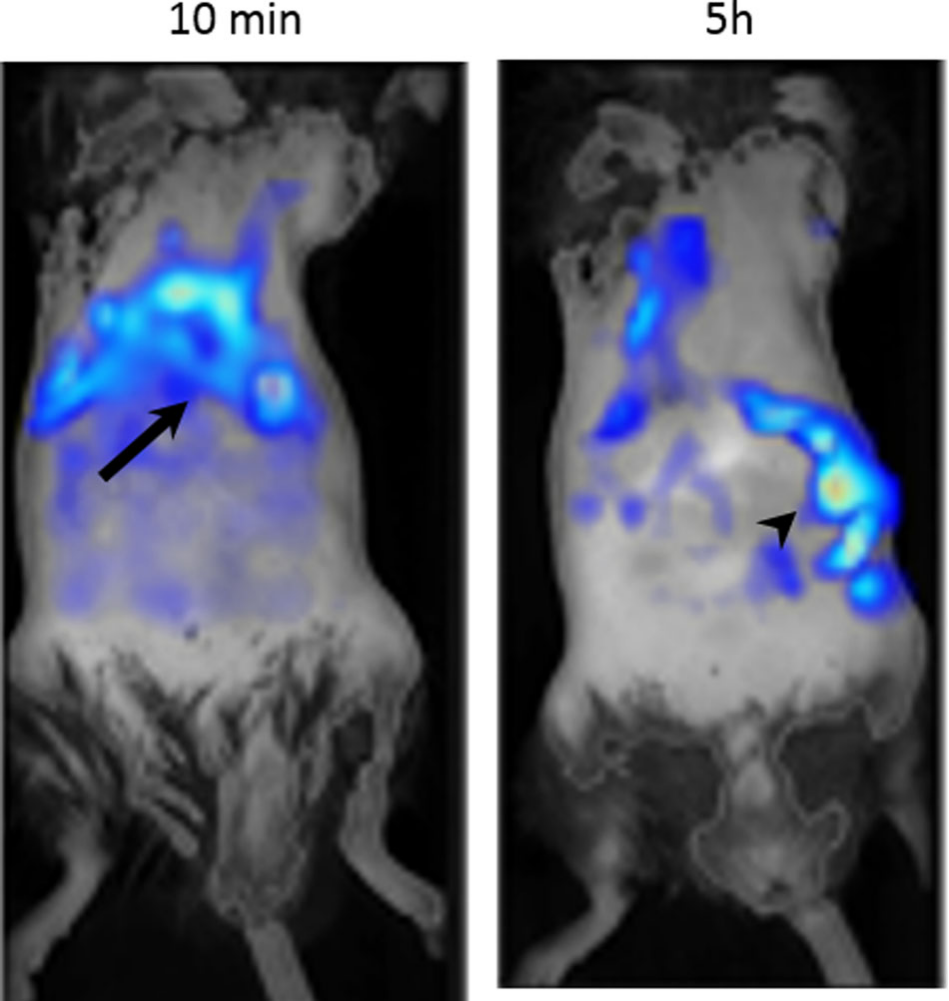
The absorption coefficients of the main tissue absorbers, water and oxy- and deoxy-haemoglobin, over 600–1100 nm. The absorption of these endogenous pigments is at its lowest from 700–900 nm, creating an ‘optical window’ for *in vivo* imaging. Reprinted with permission from Macmillan Publishers Ltd: Phan, T. G. and Bullen, A. Practical intravital two-photon microscopy for immunological research: faster, brighter, deeper. *Immunology and Cell Biology* **88**, 438–444, doi:10.1038/icb.2009.116 (2014)

### Cell labelling with probes

Many labelling techniques require cells to internalise probes, such as radionuclides, nanoparticles, paramagnetic agents, or fluorophores, which must then remain intracellular. These molecular probes can then be tracked in vivo using various different imaging techniques, depending on the probes used.

**Fig. 3:**
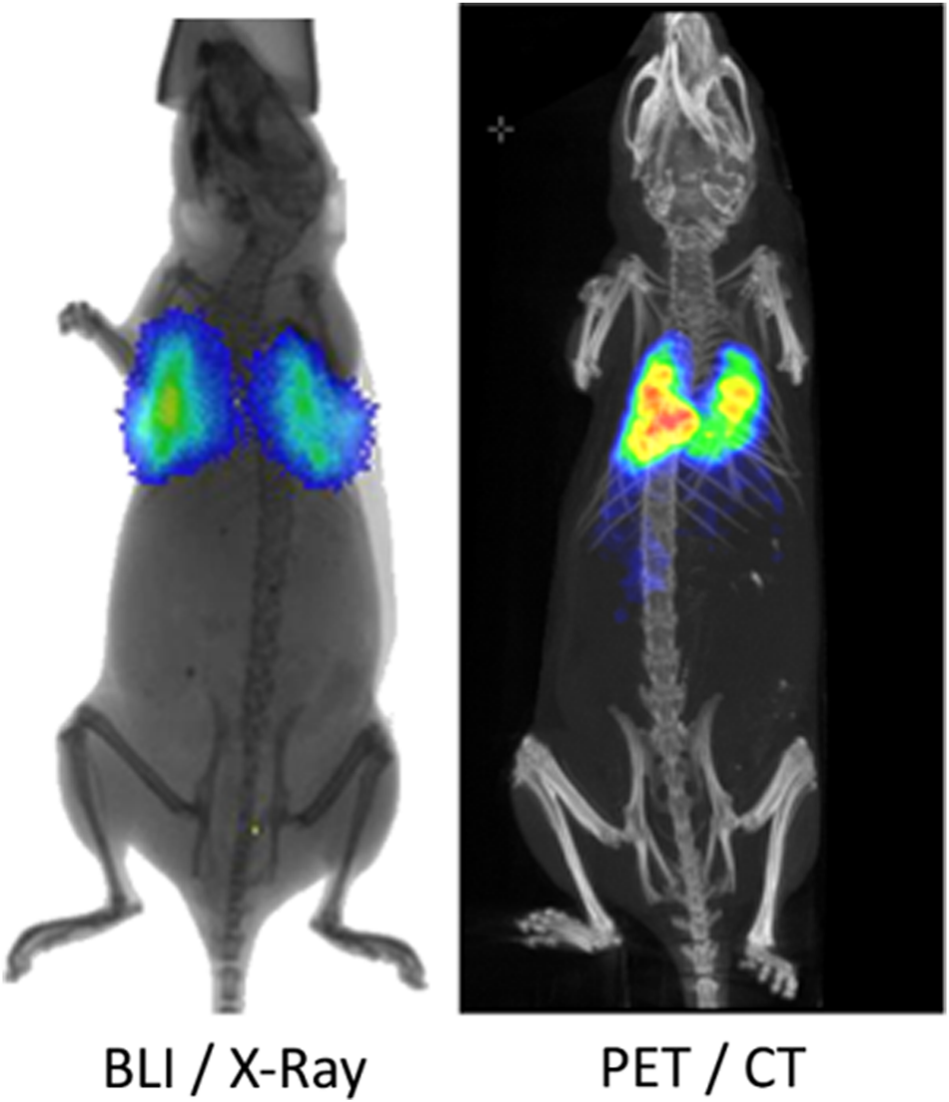
PET imaging shows the three dimensional biodistribution of intravenously injected human adipose-derived stem cells labelled with ^89^Zr-oxine. Bioluminescence imaging confirms their viability and the co-location of the cells and the radiotracer. Data generated at the Centre for Advanced Biomedical Imaging (CABI), University College London

Commonly used agents for optical imaging are fluorescent chemicals (fluorophores) including fluorescent proteins,^106^ quantum dots (QDs),^107^ and persistent luminescent nanoparticles.^108^ Optoacoustic imaging can also be used to image cells labelled with fluorophores. Paramagnetic agents, such as superparamagnetic iron oxide nanoparticles (SPIONs)^109^ and gadolinium (Gd)-based chelates,^110^ or perfluorocarbons (PFC),^111^ provide very good contrast images for use in MRI. Radionuclides are radioactive cell labelling agents used to track cells in vivo by positron emission tomography or single photon emission computed tomography, depending on the type of radionuclide used. Commonly used radionuclides include Indium-111 (^111^In), Zirconium-89 (^89^Zr) (Fig. 3),^112^ and Technetium-99m (^99^mTc).^31,113^ Optoacoustic-specific agents include small probes, such as gold nanoparticles and carbon nanotubes, which maximally absorb within the NIR wavelength range.^114^ Gold nanoparticles come in a vast range of morphologies, such as nanorods, nanotubes, nanostars, and nanospheres, and well-established synthesis procedures enable researchers to easily modify the size and morphology of these gold nanoparticles, allowing their optical properties to be tuned for specific applications.^30,114^

Most probe-labelling techniques have the advantage of producing an intense signal which can be detected by the relevant imaging modality with greater sensitivity than reporter genes, making them more effective for detecting low numbers of cells. However, all labelling probes suffer from signal dilution that occurs when the cell divides, and possible leakage of the probe from the cell of interest, resulting in false positives. Labelling probes are therefore very good for tracking cells in the short-term, but have limited potential for longer term cell tracking or tracking rapidly proliferating cells.

### Cell labelling with reporter genes

Reporter gene labelling involves genetic modification of cells to express reporter genes encoding reporter proteins, which generate imaging signals either constitutively, after an enzymatic reaction, or after binding or transport of a substrate into the cell.

Reporter genes used in optical imaging generally include bioluminescent and fluorescent reporter genes. Bioluminescent reporter genes encode a bioluminescent enzyme called luciferase that releases light energy following a chemical reaction. The most common luciferases are firefly, renilla, click beetle, or gaussia.^13,92^ As light emission relies on an enzymatic reaction, the animal must generally be administered the substrate to the enzyme prior to imaging, unless the enzyme and substrate are both expressed constitutively, such as with the bacterial luciferase operon (lux),^115^ thus precluding the need for an exogenously administered substrate. Fluorescent reporter genes, however, encode fluorescent proteins that simply require an external light source to excite the fluorophore^92^; therefore the administration of a substrate is not necessary. Fluorescent proteins that maximally absorb within the ‘optical window’ (see box 4), such as Katushka2S,^116^ suffer minimal attenuation of signal due to low tissue absorbance and are therefore more sensitive. Moreover, many fluorescent reporter genes can also be detected by optoacoustic imaging,^117^ thus allowing for multi-modal imaging. Reporter gene labelling methods used in MRI include reporter genes, such as tyrosinase,^118^ which result in T_1_ contrast following enzymatic reactions with their respective substrates, yielding an increased signal. T_2_ or T_2_*-based MRI contrast is typically generated by reporter genes, such as the transferrin receptor or ferritin, whereby an increase in the uptake or synthesis of iron or iron-bound proteins, results in a reduced T_2_ signal.^119^

Radionuclide reporter genes are detectable by PET and SPECT imaging depending on the probe utilized. Sensitivity is highly reliant on the degree of accumulation and retention of the tracer within the cell in concordance with rapid removal or wash out of unbound tracer to provide signal to noise. The herpes simplex virus type 1 thymidine kinase (HSV1-tk) reporter gene is driven by a promoter/enhancer to express the reporter protein enzyme, thymidine kinase. Thymidine kinase phosphorylates exogenously administered reporter probes, which remains restricted to the cytosol of the cell, resulting in highly specific and sensitive signals from labelled cells.^120^ Thus, radioactivity reflects HSV1-tk enzyme activity and gene expression. However, different substrates for HSV1-tk such as the acyclguanosine derivitative (9-[4-[^18^F]fluoro-3-(hydroxymethyl)butyl]guanine ([18 F]FHBG) have been shown to accumulate in cells better than others.^121^ Nuclear reporters based on ligand binding, such as the Dopamine 2 receptor (D2R)^122^ allows for one to one binding unless the receptor can be internalised. Thus, radioactivity reflects gene expression. However, this has limitations due to competitive binding with native ligand, limited levels of expression due to competition of other membrane receptors, and also some ligand-binding strategies can initiate cell signalling cascades that could lead to apoptosis or cell differentiation. Lastly, nuclear reporters based on transporter mechanisms such as the sodium-iodide symporter (NIS)^123^ or the norepinephrine transporter (NET)^124^ actively transport or pump the tracer into the cell, allowing for increased accumulation and effective signal to noise. However, the NIS system has a naturally high native expression in the thyroid and stomach, and also there is rapid efflux of the tracer from cells.

Nonetheless, reporter gene cell labelling techniques have important advantages over probes. In theory, signals generated from reporter genes are only generated from live cells, and the agent producing the signal does not transfer from labelled cells to host cells, unlike with probe-based labelling methods. However, all nuclear reporter genes, and some MRI reporter genes require the use of substrates, which in practice will produce background signals in parts of the body not containing reporter-expressing cells, due to their incomplete clearance in normal tissue. The areas of background uptake are characteristic for each substrate, meaning that reporter gene selection for a specific application can limit their use to areas with low background retention of the substrate. Importantly, and depending on the vector, due to their integration within the cell genome, reporter genes are passed on to daughter cells, thus circumventing the signal dilution that occurs with direct labelling methods and allowing for the monitoring of tumour development resulting from uncontrolled proliferation of cells. However, for longitudinal use, reporter gene techniques require cells to be stably transfected or virally transduced in order to introduce the genetic label, which poses a risk of altering the cell phenotype and possibly inducing tumourigenicity or ablating regenerative potential. The use of new genetic engineering techniques such as clustered regularly interspaced short palindromic repeats (CRISPR)/Cas that control the site of genetic modification can however reduce the risk of disrupting the coding sequences of native genes.^125^

### The optical window

**Fig. 4:**
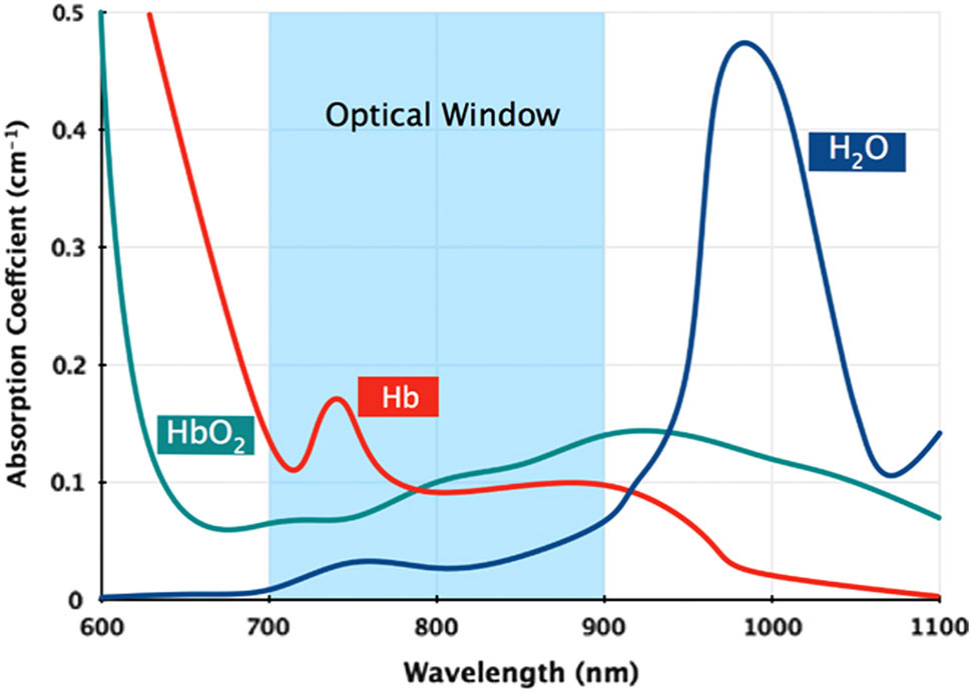
The absorption coefficients of the main tissue absorbers, water and oxy-haemoglobin and deoxy-haemoglobin, over 600–1100 nm. The absorption of these endogenous pigments is at its lowest from 700–900 nm, creating an ‘optical window’ for in vivo imaging. Reprinted with permission from Macmillan Publishers Ltd: Phan, T. G. and Bullen, A. Practical intravital two-photon microscopy for immunological research: faster, brighter, deeper. *Immunology and Cell Biology* **88**, 438–444, doi:10.1038/icb.2009.116 (2014)

Biological tissues contain several different light-absorbing molecules, such as haemoglobin, melanin, fat, and water, each of which absorb maximally at characteristic wavelengths. Water and oxy-haemoglobin and deoxy-haemoglobin are the major light absorbers in the tissues of animals, with the exception of animals that exhibit black skin pigmentation, which also have strong absorption by melanin. The 700–900 nm wavelength range is known as the ‘optical window’ or the ‘imaging window’ as it is in this region that water and haemoglobin have their lowest absorption coefficient (Fig. 4). Therefore, light can penetrate most deeply within this wavelength range.^126^

## Preclinical imaging approaches to evaluate cell-based RMTs

### Cell biodistribution

It is essential to be able to track cells following their administration and engraftment into the host, and imaging methods can be used to answer some key questions regarding cell biodistribution. For example, where do the cells go when they are administered, particularly if they are administered systemically? Following systemic injection, do the cells eventually reach the target organ, and how long does it take them to do so? Do the cells integrate within non-target organs? Alternatively, if the cells are administered directly to the target organ, was the injection successful? Do the cells stay within the target organ, or do they migrate elsewhere over time?

Previously, these questions would have been answered by sacrificing multiple animals at several different time points and detecting the transplanted cells via histological techniques. However, by using imaging modalities, labelled cells can be tracked over time in individual animals.^38^ Bioluminescence imaging is one of the most useful modalities for monitoring cell biodistribution, as whole body images of multiple animals can be generated simultaneously in a matter of seconds, while detecting as few as 10 cells.^39^ This modality can be used to monitor the immediate biodistribution of cells following administration, and is particularly useful for confirming a successful injection,^40^ in addition to tracking cell biodistribution over time (Fig. 2). Most importantly, due to the requirement of an active ATP metabolism for light production in cells expressing firefly luciferase, this reporter provides a remote and highly-sensitive readout on whether the cells are alive or not. Using bioluminescence imaging, Yi Tang et al. tracked luciferase^+^ neural progenitor cells for up to 4 weeks as they migrated through the parenchyma of the brain from the injection site into a brain tumour.^38^ However, a limit to optical imaging is its depth limitation and poor spatial resolution^13^ and the fact that it is mostly restricted to 2D planar imaging. This means that although the general biodistribution of cells can be imaged, it can be difficult to tell exactly which organ the cells are located in, and it is not possible to monitor distribution within specific organs. 3D optical imaging is possible,^41^ but it relies on a pre-determined anatomical template, which may not accurately match with the individual animal.

MRI and optoacoustic imaging are both able to provide much higher spatial resolution,^42, 43^ and so can provide more detailed information regarding cell location, and in some cases can also allow for quantification of approximate cell number. Nam et al. used ultrasound-guided optoacoustic imaging to track mesenchymal stem cells (MSC) labelled with gold nanotracers for 1 week following implantation.^44^ In vitro studies from this group suggested that the amplitude of the optoacoustic signal was directly related to the concentration of gold nanotracers, allowing for reasonable confidence in the quantification of cell numbers in vivo.^44^

Nuclear imaging, e.g., PET and SPECT, are highly sensitive imaging modalities that can be used to track cell biodistribution in vivo over the short-term and long-term, based on radionuclide probe or genetic reporters, respectively. The PET probe, [^18^F]-2-fluoro-2-deoxy-d-glucose (^18^F-FDG), is a widely used biomarker of cancer because it measures glucose metabolism, which is increased in cancer cells. ^18^F-FDG can also be used to monitor the immediate biodistribution of injected stem cells,^45^ but long-term tracking with ^18^F-FDG is not possible due to its short half-life (110 min). Cells can be tracked for up to a few days using isotopes with longer half-lives, such as copper-64 (^64^Cu), ^111^In, and ^89^Zr,^46, 47^ but longer-term tracking requires the use of genetic reporters, such as the HSVtk reporter that has been used to monitor the biodistribution of progenitor cells for over 5 months in a porcine model of myocardial injury.^48^ HSV1-tk has been the most widely used nuclear reporter system to have been used in a range of regenerative cell types in vivo,^48^ it is the only reporter gene to have been clinically translated for the purpose of monitoring T cell immunotherapies to cancer.^49^ As previously mentioned, immunogenicity of a non-human derived reporter protein has been a problem for clinical translation. To overcome this limitation, a human mitochondrial thymidine kinase type 2 (hTK2) has been proposed.^50^ The further development of nuclear reporter systems for regenerative cells has therefore been based on human derived genes that have been previously worked up in other cell lines, such as the as the D2R,^51^ and NIS^52^ systems. Although this counters any immunogenicity concerns, the limitation is that there is increased background uptake of the reporter probe natively expressing tissues within the body. The sensitivity and resolution is thereby dependent on the biodistribution of the reporter probe. A shorter half-life is preferred for radionucleotide probes for reporter systems as this allows for multiple imaging acquisitions over a longitudinal time frame such as in the case of the HSVtk reporter which has been used to monitor the biodistribution of progenitor cells for over 5 months in a porcine model of myocardial injury. Alternative approaches that involve the systemic administration of cell targeting probes can also be used to monitor cell biodistribution and/or provide information on cell phenotype. For example, ^64^Cu (bound to arginine-glycine-aspartic (RGD) tetramer conjugated with the macrocyclic chelator 1,4,7,10-tetraazacyclododecane-*N*,*N*′,*N*″,*N*‴-tetraacetic acid (DOTA)) was used to target αvβ3 integrin, in order to detect whether human embryonic stem cells^53^ formed teratomas. However, a downside to this approach is the lack of specificity.

**Fig. 5:**
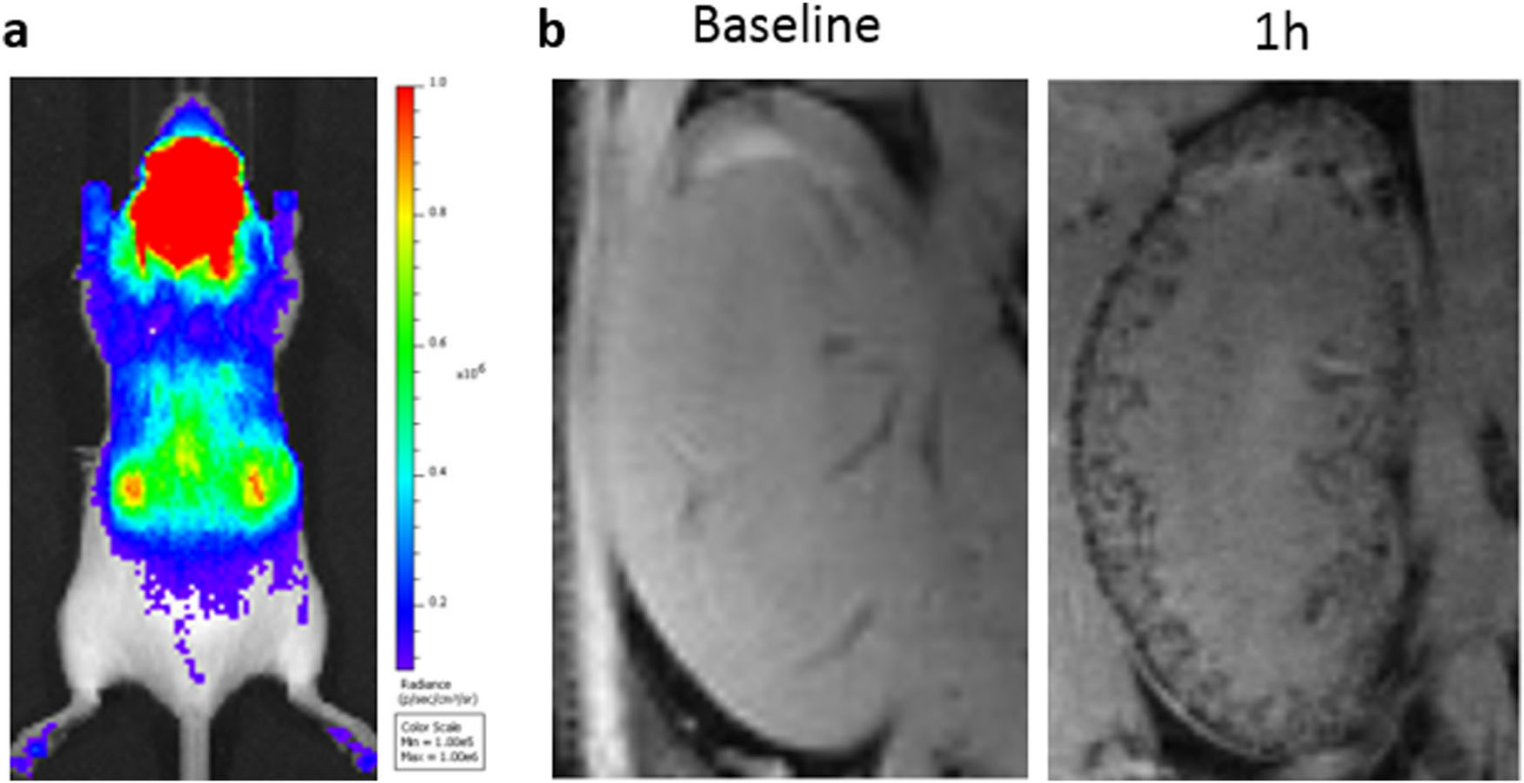
Multi-modal imaging of Luciferase^+^/SPION^+^ stem cells administered to the left cardiac ventricle. **a** BLI gives a fast confirmation of successful IC injection, and gives an approximate location of cells, but lacks organ-specific information. **b** MR imaging of the kidneys before and after the administration of SPION-labelled stem cells reveals that SPION-labelled cells are within the cortex of the kidney. Data generated at the Centre for Preclinical Imaging, University of Liverpool

Dual-labelling of cells with both probes for short-term tracking and reporter genes for long-term tracking might satisfy requirements for both highly sensitive immediate biodistribution, as well as longitudinal tracking for tumour monitoring. For example, cells could be transduced with luciferase for bioluminescence imaging, providing high sensitivity but poor spatial resolution, and also labelled with SPIONs for MR imaging, permitting the intra-organ biodistribution to be evaluated with the excellent spatial resolution of MRI (Fig. 5).

Alternatively, cells could be labelled with a single reporter gene which allows for dual-modal imaging. Patrick et al. described a reporter gene system based on the organic anion transporting protein (Oatp1a1), which mediated the uptake of two contrast agents for MRI and SPECT imaging, gadolinium- (Gd) ethoxybenzyl-diethylenetriamine pentaacetic acid (EOB-DTPA) and ^111^In-EOB-DTPA, respectively.^54^ Oatp1a1-expressing cells were implanted in the flanks of mice, and after systemic administration of contrast agent, could be imaged longitudinally using both MRI and SPECT, thus combining the advantages of both modalities: *i.e*., the high spatial resolution of MRI, and the sensitivity of SPECT. Further, their sensitivity of detection with bioluminescence was enhanced, due to Oatp1a1’s ability to increase uptake of the substrate.^55^ Unlike labelling probes such as SPIONs or radionuclides used to track cells with MRI and SPECT, this reporter gene system can be used to monitor dividing cells over time, and does not suffer from signal dilution.

Ngen et al. have described a dual contrast system comprised of SPIONs and gadolinium chelates, which generate opposing contrast signals and allow for the differentiation between live and dead cells.^56^ When both contrast agents are present in live cells, the strong T_2_ signal from the SPIONs quenches the T_1_ contrast from the gadolinium chelates. However, when cells die, the gadolinium chelates are released and diffuse away from the SPIONs, allowing the T_1_ signal to be detected in the region surrounding the dead cells.^56^

When aiming to track cells over a long period, the cell label should be chosen carefully. Some cell labels, while very sensitive, cannot be detected after a certain period, due to either their chemical degradation or radioactive decay. As a general rule, labelling probes are unsuitable for long-term cell tracking, unless the cells are non-proliferating; for instance, it has previously been shown that SPION-labelled neural progenitor cells can be tracked in vivo for several weeks with MRI as these cells do not proliferate following their differentiation.^57^

### Tumourigenicity

A well-known safety concern of cell-based RMTs is the potential for tumour formation by the engrafted cells.^42^ Stem cells have the capacity for self-renewal and as such, may proliferate after administration to form tumours.^58^ Pluripotent stem cells (PSCs) pose a particular risk due to their tendency to form teratomas and/or teratocarcinomas. However, with PSC-based therapies, it is not the undifferentiated PSCs themselves that are administered, but rather, their more differentiated derivatives; for instance, PSC-derived retinal pigment epithelial cells are currently being tested in the clinic for their potential to treat age-related macular degeneration (ARMD).^59^ The main concern with such therapies is the risk of tumour formation in the host by small numbers of contaminating undifferentiated PSCs which might be present within the administered population.

**Fig. 6:**
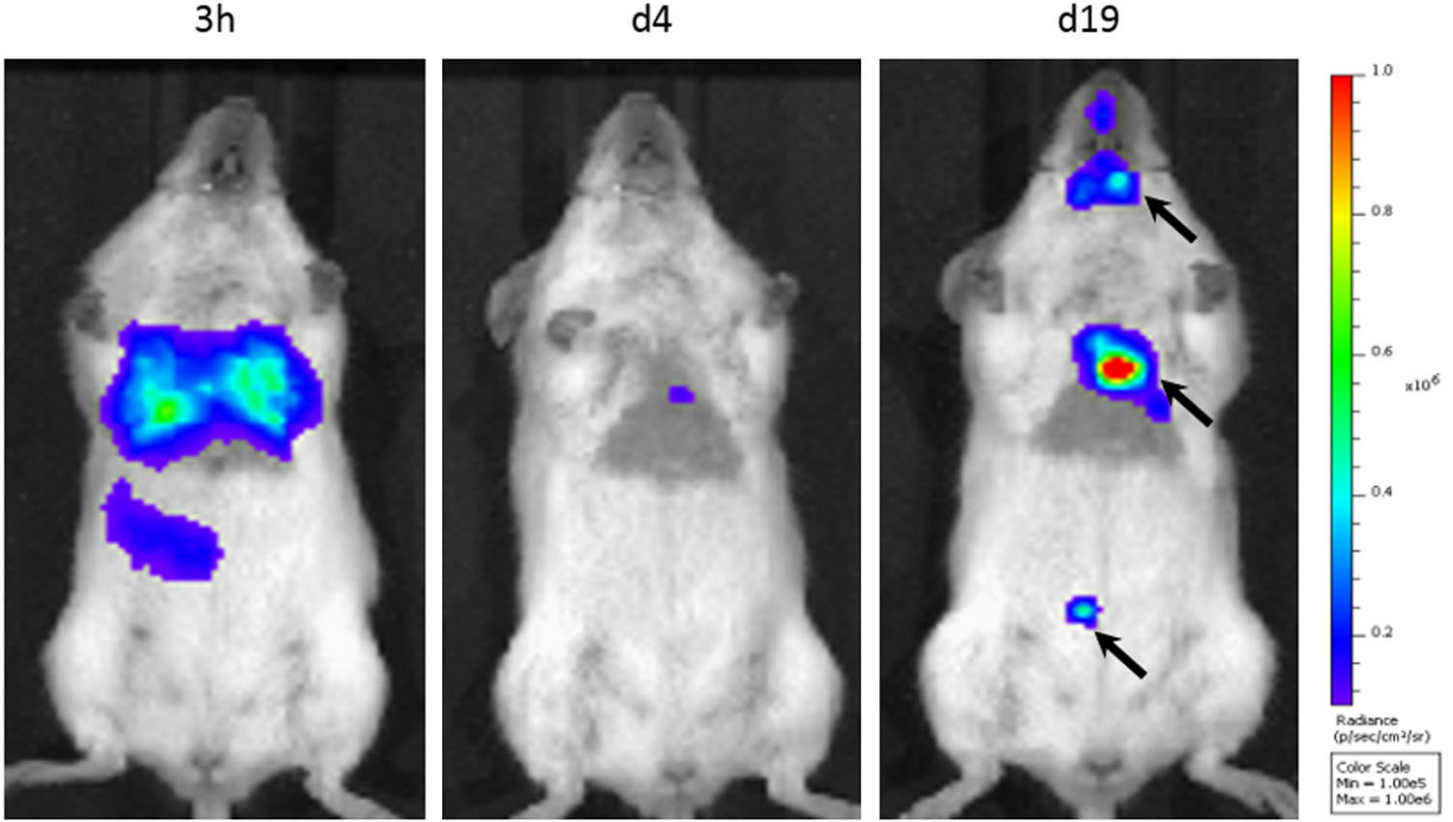
BLI of Luciferase^+^ mouse kidney stem cells after intracardiac administration. BLI highlights the need for longitudinal imaging, as the signal from cells can decrease initially as cells die, but tumours (arrows) may form at later time points. Data generated at the Centre for Preclinical Imaging, University of Liverpool

As it is difficult to completely exclude this possibility, even when using sensitive techniques such as quantitative PCR, it is important to assess the risk of tumourigenicity in animal models prior to commencing clinical trials. This is most easily done using constitutively expressed reporter genes, because if integrated into the genome, the reporter genes will be passed onto the daughter cells when the original cells divide.^36^ Thus, if the cells proliferate following transplantation, there will be an increase in signal intensity, enabling tumour growth to be monitored in vivo^36^ (Fig. 6).

In addition to the administered cells themselves forming tumours, it is also possible that they could promote the growth of endogenous tumours that are already present in the host; this has previously been demonstrated following the administration of MSCs into immune-compromised mice.^60^ Sensitive techniques are required to detect such tumours, the most common being ^18^F-FDG-PET, which can be used in both the preclinical and clinical setting. Various approaches based on optoacoustic imaging are also being developed, including enhanced haemoglobin contrast that is a feature of highly vascularised tumours^61^ and uptake of the NIR dye, indocyanine green, which passively accumulates in tumours.^62^ Furthermore, specific tumour imaging can be achieved by conjugating optoacoustic probes such as gold nanoparticles to antibodies specific to particular cancer cell antigens.^63^

### Immunogenicity

Cell-based RMTs derived from allogeneic sources have a high risk of being immunogenic.^64^ Even autologous cells derived from the patient have the potential to evoke an immune reaction when transplanted back into the host, as in vitro culture conditions may induce genetic, epigenetic, and phenotypic changes within the cells.^62,65^ Prior to translating a cell therapy to the clinic, it is important to try to determine the immunogenic potential of the human cells as thoroughly as possible. This cannot be done using animal models alone due to the inherent differences between animal and human immune systems,^61^ and a combination of in vitro and in vivo studies is required. Nevertheless, adoptive transfer and subsequent imaging of immune cells may provide a means by which to monitor an animal’s immune response to cell therapy over time.

A fluorescent lipophilic dye, 1,1-dioctadecyltetramethyl indotricarbocyanine iodide (DiR), has been used previously to label and track adoptively transferred macrophages^66^ and T-lymphocytes,^67^ thus allowing imaging of the immune reaction by proxy of exogenously administered cells. Eisenblätter et al. administered DiR-labelled macrophages intravenously to a mouse model of cutaneous granuloma, to non-invasively monitor the early inflammatory response to subcutaneously implanted lipopolysaccharide.^66^ This approach could be applied to image the inflammatory response following the administration of an RMT. Further, a range of fluorescent probes can be directly administered to animals to image sites of inflammation using fluorescence imaging.^68^ Haney et al. used the XenoLight RediJect Chemiluminescent Probe, by Perkin Elmer, to image inflammation levels following macrophage-mediated therapeutic drug delivery in a mouse model of Parkinson’s disease.^68^ Alternatively, Faraj et al. recently used MRI to non-invasively track SPION-labelled macrophages to sites of inflammation in a mouse model of chronic obstructive pulmonary disorder.^69^

Optoacoustic imaging can also be used to monitor the immune response to administered cells. Ricles et al. labelled cells with both gold nanorods and gold nanospheres, which have different absorption spectra, thus allowing the two labels to be distinguished separately in vivo.^70^ The peak absorption of gold nanospheres is changed when they are endocytosed by macrophages, allowing the differential identification of signals coming from live cells labelled with gold nanorods, and those coming from endocytosed cells labelled with the now-visible gold nanospheres. Using this method, Ricles et al. could monitor the viability of administered therapeutic cells in vivo and the rate of tissue macrophage infiltration over time. Additionally, by conjugating gold nanorods to antibodies for inflammatory cytokines, inflammation can be detected in vivo using optoacoustic imaging.^71^

However, for many reasons, we may not be able to fully understand the immunogenic potential of cell therapies during preclinical testing. Many products in preclinical research are xenogeneic in the animal model, and differences in the animal and human cellular product mean that the animal equivalent is not fully predictive of the potential for immunogenicity in humans. Moreover, for preclinical testing of a xenogeneic product, the animals will either be immunocompromised or suppressed, which is not necessarily the case in the clinical setting. Nonetheless, preclinical imaging can aid in understanding aspects of the interaction of the therapy with the immune system, and may be able to inform the selection of RMTs with a lower potential for immunogenicity.

### Monitoring cell fate

An important aspect of monitoring the safety and efficacy of RMTs involves understanding the fate of the cells following administration. This is especially important for therapies based on progenitor cells, where amelioration of disease requires the cells to differentiate in vivo to one or more specialised cell types; examples of such therapies include pluripotent stem cell (PSC)-derived dopaminergic neuroblasts and PSC-derived oligodendrocyte precursor cells for the treatment of Parkinson’s disease^72^ and multiple sclerosis,^73^ respectively. Differentiation status can be assessed using cell-type-specific promoters to drive the expression of a reporter gene. By combining a cell type-specific reporter with a constitutively expressed reporter, it would be possible to monitor the viability and biodistribution of all cells within the administered population, and determine the proportion of cells which undergo differentiation.

Recently, Ahn et al. demonstrated the use of a dual reporter gene encoding both renilla and firefly luciferases, which can be imaged independently using bioluminescence imaging.^74^ In stably-transduced embryonic stem cells, the expression of renilla luciferase was driven by the Oct4 promoter, and the expression of firefly luciferase was driven by the ubiquitin promoter, allowing the non-invasive monitoring of stem cell differentiation in vivo.^74^

Imaging can be a vital tool for monitoring the effect of interventions to enhance the survival of administered cells in vivo. Yang et al. used bioluminescence imaging to assess the survival of adipose-derived stem cells (ADSCs) injected along the infarct border in a rat model of myocardial infarction.^75^ Some rats were administered ADSCs alone, while others were administered ADSCs in combination with an injectable fibrin scaffold to aid cell survival. BLI at 4 weeks after cell transplantation showed that the addition of the fibrin scaffold significantly improved the survival of transplanted cells.^75^ 3D biodegradable scaffolds are very important in some RMTs, as they provide the therapeutic cells with the structural support to proliferate and differentiate appropriately. The scaffold is usually designed to break down after a certain period of time, and it is important to be able to monitor the fate of the scaffold over time. Nam et al. recently demonstrated the use of multimodal imaging for monitoring and quantifying the degradation process, in addition to monitoring the labelled therapeutic cells that were seeded on the scaffold.^76^

### Efficacy of RMTs

**Fig. 7:**
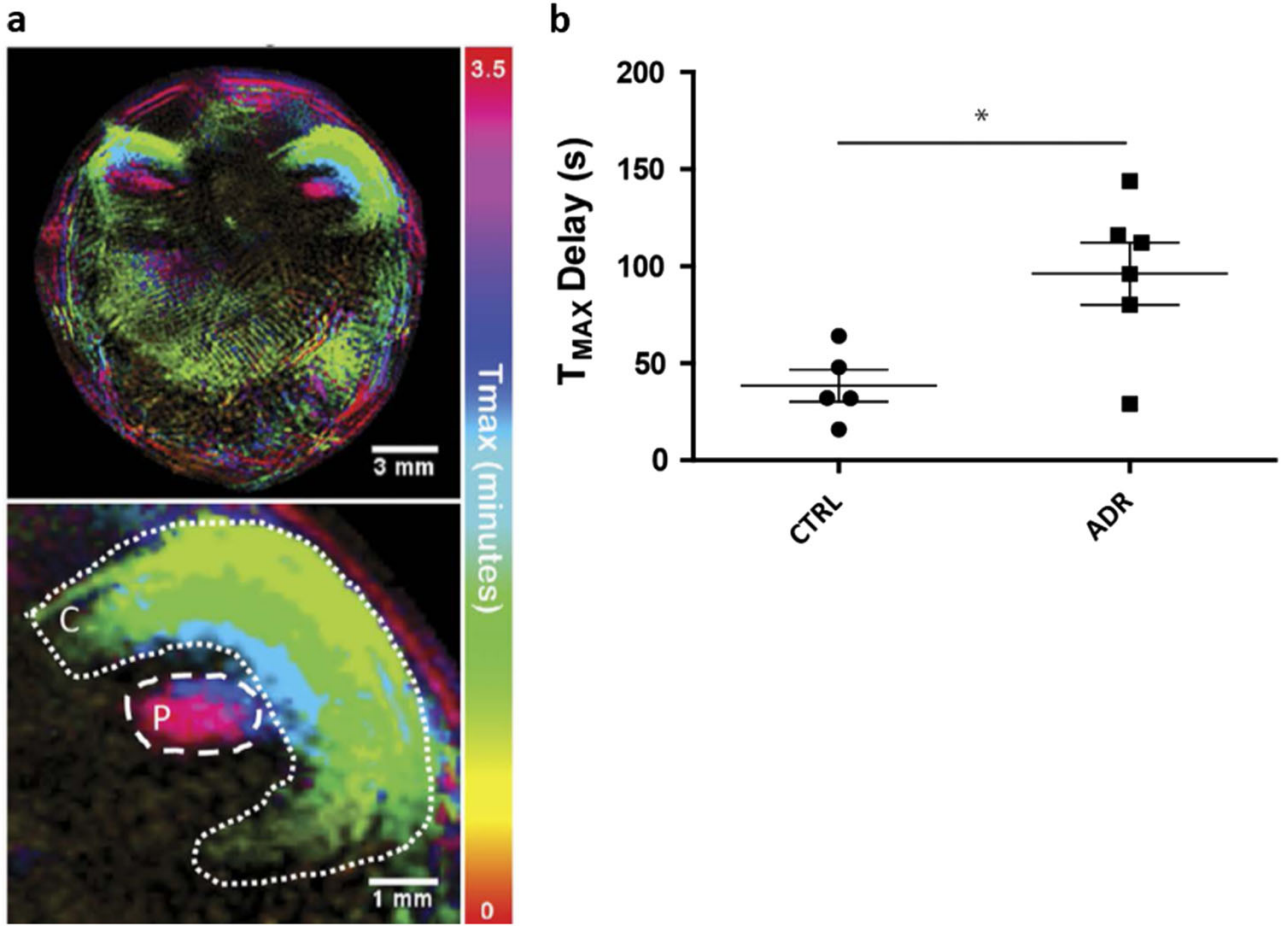
Photoacoustic (multispectral optoacoustic tomography, MSOT) imaging of kidney clearance kinetics. **a** Temporal colour map indicates the time it takes for a near infrared dye to clear through different regions of the kidney; cortex (C) and pelvis/papilla (P). **b** Quantification of the clearance kinetics of a NIR dye through the kidneys of control mice (CTRL) and mice with kidney injury (ADR), demonstrating that photoacoustic imaging can be used to measure organ function. Reprinted under the Creative Commons CC-BY license from Scarfe et al. *Scientific Reports* **5**, doi: 10.1038/srep13601 (2015)

In addition to monitoring cell biodistribution and fate, in vivo imaging technologies allow the assessment of organ function, and can therefore be used to monitor the efficacy of RMTs. By imaging organ function at baseline, after induction of injury, and after therapeutic intervention, it is possible to monitor each individual animal’s response to therapy. For instance, optoacoustic imaging is excellent for monitoring organ function. The clearance of exogenously administered dyes such as ICG and IRDye800 CW, which are specifically cleared through the liver^26^ and kidney^77^ (Fig. 7) respectively, allow assessment of organ function. Optoacoustic imaging can also be used to assess oxygenation status,^78^ brain function, such as resting state functional connectivity,^79^ and angiogenesis.^80^

Experimental imaging studies can be designed to perform cell tracking and efficacy assessment in the same animal, and during the same imaging session. Thus, individual responses to therapy can be correlated with cell biodistribution and other cell tracking data, such as proliferation and differentiation status, increasing the amount of information gained from each animal. Recently, Nam et al. demonstrated the value of optoacoustic imaging in regenerative medicine to assess the severity of injury in a cutaneous burn model, while simultaneously tracking gold nanorod-labelled ADSCs.^81^

MRI can also be used to image organ function and tissue regeneration. For example, Freeman et al. used MRI to assess the efficacy of MSCs administered directly into the intervertebral disc in a study of degenerative disc disease in sheep.^82^ Using MRI, the authors measured disc height and disc degeneration at multiple time points, allowing the response to therapy for each individual animal to be monitored over time. It is important to note that apart from anatomical imaging, MRI offers a range of advanced techniques that are currently used pre-clinically and clinically for the evaluation of disease progression and response to therapies. Those include, for example, diffusion weighted imaging, perfusion imaging and MR spectroscopy, all of which are now well established or under consideration for monitoring diseases of the heart,^83^ liver,^84^ kidney ^85^ and brain^86^ and cartilage,^87^ among others. Given the importance of these techniques, it is expected that many of them will be also applied for the assessing RMT efficacy.

### Mechanisms of action

It is not enough simply to know that a cell type is efficacious in treating a disease. Before clinical translation, it is important to understand why the therapy appears to work, and its mechanism of action. Do the cells need to integrate within the organ of interest in order to have an effect? Do the cells even need to be present in the organ of interest, or do they have endocrine or paracrine effects which result in a resolution of the disease? If cell-derived factors rather than the cells themselves are responsible for promoting regeneration, then these could potentially be isolated and used to develop a cell-free therapy.

For instance, recent studies have shown that following intravenous injection of MSCs^88^ or kidney-derived cells^89^ into rodents with kidney disease, significant therapeutic effects were observed despite the fact that the cells were entrapped in the lung and did not engraft in the kidney. Imaging can also be used to optimise the ideal dosing conditions for maximum efficacy of a cell therapy, including the route of administration, number of cells required per dose, and timing of dosing.^40, 90^

As released in guidelines from the European Medicines Agency, the mechanism of action is also important to define a “potency assay”, which should be used at the release of the finished product before clinical application to show that the biological product will be able to perform the intended clinical effect. This potency assay should not only show that the cells are viable and can be identified as e.g., MSCs, but also include a functional assay. The assay demonstrating the biological activity should be based on the intended biological effect which should ideally be related to the clinical response.^91^

## Summary

Preclinical imaging is a valuable tool for the assessment of various aspects of the safety and efficacy of RMTs prior to clinical translation. However, effective use of imaging technologies and cell labels requires full understanding of their limitations, as well as their potential. An important weakness to consider is the limit of detection, with particular emphasis on the number of cells that can realistically be detected with each modality/cell label combination. The key to successful use of imaging technologies is understanding what is achievable and what is not, and full acknowledgement of these limitations will enable the data that is generated to be put into clinical context. This will allow the consideration of subsequent alternative methods, such as traditional pathology assessment of the animals, or combination with alternative, complementary imaging technologies.

Multimodal imaging is central to effective evaluation of RMT safety and efficacy. No single imaging modality is ideal; all are associated with their own intrinsic strengths and weaknesses and by combining two or more modalities, they can complement one another to provide the maximum amount of information from a single animal. Key to this is therefore dual- or triple-labelling of the cells of interest for their visualisation by multiple imaging modalities, thus gaining more information from each animal than could be achieved with a single imaging modality.^54, 56, 70^ It is, however, essential that adequate in vitro analyses are performed prior to in vivo application, to ensure that all cell labels are complementary with one another, and do not have adverse effects on cell health or phenotype. Multimodal imaging approaches to monitor cell biodistribution, cell fate, therapeutic response etc., can vastly reduce the number of animals required for RMT safety and efficacy studies, as several different parameters can be assessed longitudinally in the same group of animals, without the need to sacrifice multiple animals at various time points. Multimodal imaging therefore supports the principles of the 3Rs (Reduction, Refinement, Replacement) by reducing the total number of animals required for such studies. Imaging technologies are essential in the comprehension of the mechanisms of action and potential safety issues of RMT and thus will allow a more accurate evaluation of the risk:benefit ratio of these therapies. These technologies will be essential (or a key player) to move the RMT to clinical development.
